# The Characteristics of Blood Glucose and WBC Counts in Peripheral Blood of Cases of Hand Foot and Mouth Disease in China: A Systematic Review

**DOI:** 10.1371/journal.pone.0029003

**Published:** 2012-01-03

**Authors:** Yuyun Li, Runan Zhu, Yuan Qian, Jie Deng

**Affiliations:** Laboratory of Virology, Capital Institute of Pediatrics, Beijing, China; Institut Pasteur, France

## Abstract

**Background:**

Outbreaks of Hand Foot and Mouth Disease (HFMD) have occurred in many parts of the world especially in China. We aimed to summarize the characteristics of the levels of blood glucose and white blood cell (WBC) counts in cases of HFMD in Mainland China and Taiwan, using meta-analysis based on systematic review of published articles.

**Methods:**

We systematically reviewed published studies, from the MEDLINE and WANFANG Data, about the levels of blood glucose and WBC counts in cases of HFMD until 15^th^ June 2011, and quantitatively summarized the characteristics of them using meta-analysis.

**Results:**

In total, 37 studies were included in this review. In Mainland China and Taiwan, generally, the average level of blood glucose, the prevalence of hyperglycemia, WBC counts and the prevalence of leukocytosis increased with the severity of the illness. There was no significant difference in the prevalence of leukocytosis between ANS (autonomic nervous system dysregulation)/PE (pulmonary edema) group and CNS (central nervous system) group, and in the average level of blood glucose between healthy controls and mild cases of HFMD. WBC counts in cases infected by EV71 were less than those in cases infected by CA16.

**Conclusions:**

our analyses indicated that blood glucose and WBC counts increased with the severity of HFMD disease, which would help doctors to manage patients efficiently.

## Introduction

Hand foot and mouth disease (HFMD), which was first reported in New Zealand in 1957, is an unremarkable illness that commonly occurs in young children, most of whom are younger than 5 years old. Two closely related viruses, coxsackievirus A16 (CA16) and enterovirus 71 (EV71), which belong to the *Enterovirus* genus of the *Picornavirus* family, and usually co-circulate during HFMD outbreaks [1]–[3], have been identified as the most frequent pathogens of HFMD, and other enteroviruses, including CA2, CA4, CA5, CA7, CA9, CA10, CA16, CB1, CB2, CB3, CB4 and CB5 can also cause HFMD. In the most common manifestation which gives the syndrome its name, children typically present with vesicular exanthema on the soles of their feet, the palms of their hands and in their mouths, causing discomfort and feeding difficulties. However, severe health consequences or deaths may occur owing to complications such as encephalitis, aseptic meningitis, acute flaccid paralysis, myocarditis, autonomic nervous system dysregulation (ANS) and pulmonary edema (PE) or hemorrhage.

In recent years, outbreaks of HFMD have increased and more and more severe cases have appeared, especially in China [4], which has posed a persistent and severe public health problem. Severe HFMD cases have many characteristics in laboratory diagnosis. Hyperglycemia and leukocytosis are two important characteristics in severe cases of HFMD, and blood glucose and white blood cell (WBC) counts change with the severity of the disease, which have been addressed by multiple studies such as those reported by Mao et al [5] and Yu et al [6], and the results are not always consistent. Meta-analysis and systematic review can solve these disputes. To our knowledge, these data have not been systematically evaluated and reported in English. The objective of this study was to summarize the data and evaluate the characteristics of these indexes in patients of HFMD using a meta-analysis of published articles.

## Materials and Methods

### Literature search

Studies addressing blood glucose and/or WBC counts in cases of HFMD were identified by searching for articles in the MEDLINE database and WANFANG Data until 15^th^ June, 2011. WANFANG Data is an affiliate of Chinese Ministry of Science & Technology and has been the leading information provider in China since 1950 s. With a wide range of database resources and value-added services, WANFANG Data has become a gateway to understand Chinese culture, medicine, business, science, etc.

Various combinations of the English terms as well as the corresponding Chinese terms “enterovirus”, “enterovirus 71”, “EV”, “EV71”, “hand foot and mouth disease”, “HFMD”, ‘mild cases”, “severe cases”, “blood glucose”, “hyperglycemia”, “leukocyte”, “white blood cell counts”, “WBC counts” and “leukocytosis” were used to screen for potentially relevant studies. Additional studies were also identified using cross-referencing.

### Inclusion and exclusion criteria

Studies addressing blood glucose and/or WBC counts, healthy controls and mild cases of HFMD, and/or mild cases and severe cases of HFMD, and/or cases with central nervous system (CNS) and cases with autonomic nervous system dysregulation (ANS) and/or pulmonary edema (PE) [4] and/or cases infected by EV71 (EV71) and cases infected by CA16 (CA16) were included. If the study was reported in duplicate, the article published with more cases was included. Normally, the levels of blood glucose range from 3.89 mmol/L to 6.11 mmol/L [7].When the level of blood glucose is more than 6.11 mmol/L or random blood glucose level is more than 11.1 mmol/L, it is defined as hyperglycemia. In regular health checkup, WBC counts in peripheral blood are 4∼10×10^9^ cells/L [8] and it is defined as leukocytosis when WBC counts are more than 10×10^9^cells/L. Sometimes hyperglycemia and leukocytosis were defined in a different way. In the included studies, the screening of blood glucose was usually performed by Automatic Biochemical Analysor or glucometer and WBC counts were performed by Hematology Analyzer on admission. A diagnosis of HFMD was made based on typical clinical symptoms, abiding by the handbook of treatment for HFMD issued by the Ministry of Health of the People's Republic of China. According to the handbook, mild cases of HFMD are defined as being present with fever, vesicular exanthema on the palms of their hands, on the soles of their feet and in their mouths, while severe cases are defined as HFMD cases complicated with encephalitis, aseptic meningitis, acute flaccid paralysis, myocarditis, ANS, PE, pulmonary hemorrhage, severe sequelae or even death. Review articles, studies in languages other than English or Chinese, studies without mild cases and severe cases of HFMD or healthy control and mild cases of HFMD or CNS and ANS and/or PE or cases infected by EV71 and cases infected by CA16, studies with mild cases of HFMD only or severe cases of HFMD only, the levels of blood glucose and/or WBC counts not being present as mean and standard deviation (x±s), the level of glucose and/or WBC counts in cerebrospinal fluid and case report, were excluded.

### Data extraction and Statistical analysis

For all included studies, we extracted the following data from original publications: first author and year of publication, sample size and age of the participants, male to female ratio, the diagnosis criteria of HFMD, the diagnosis criteria of hyperglycemia and/or leukocytosis, the assay method of blood glucose and/or WBC counts, the number of positive cases with hyperglycemia and/or leukocytosis of HFMD, the positive rate of cases with hyperglycemia and/or leukocytosis of HFMD, the levels of blood glucose and/or WBC counts (x±s), location rural/urban, absence of concomitant infections, ethnicity and the assay time (Table S1, Table S2, Table S3, Table S4 and Table S5). In the included studies, all the cases were collected from hospitals. For some studies, events had to be calculated from the reported data.

For each study, the odds ratio (OR) or mean difference (MD) of the most adjusted model was used to estimate a pooled OR or MD, which was obtained by the combinations of study-specific ORs or MDs using fixed effects model (Mantel-Haenszel) [9] or random effects model (DerSimonian and Laird) [10]. Random effects model was more appropriate when heterogeneity appeared. The significance of the pooled OR or MD was determined by the Z test (P<0.05). Chi square based Q test was performed to assess the between-study heterogeneity, which was considered significant for P<0.10. The I^2^ statistic for each analysis was calculated as a measure of the proportion of the overall variation that was attributable to between-study heterogeneity [11], which was independent of the number of the studies in the meta-analysis. I^2^ took values form 0 to 100% with higher values denoting greater degree of heterogeneity. Stratified analyses were performed according to age.

All analyses were applied with Review Manager Software version 5.0 (Cochrane Collaboration, Copenhagen, Denmark). The software Stata (version 10.0) was applied for meta-regression analysis to investigate the potential interactions of age and gender on blood glucose level (P<0.05).

## Results

**Figure 1:**
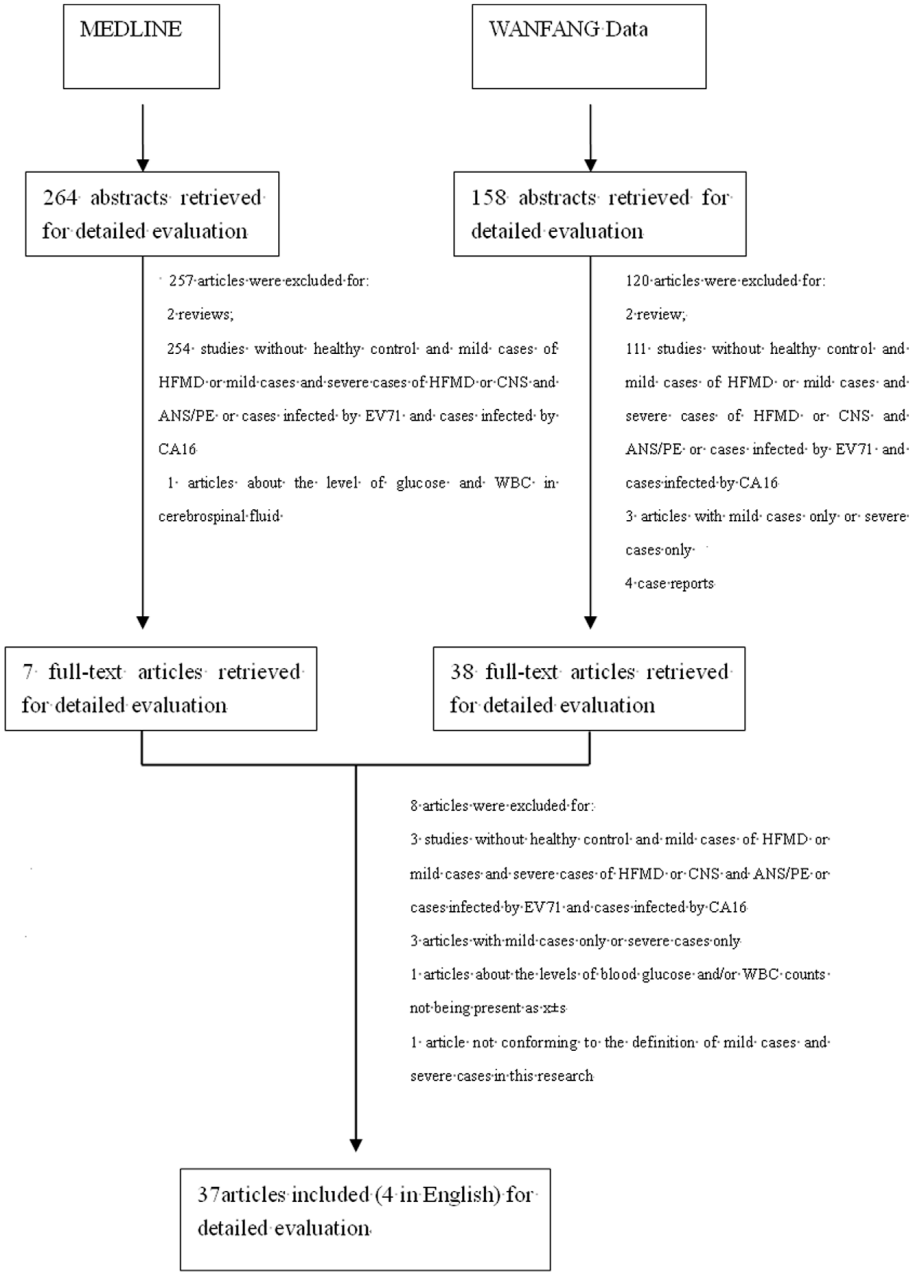
Flow diagram of study identification.

As shown in Fig. 1, a total of 422 articles published in Chinese or English were identified. After excluding 4 review papers and 377 original articles without data about healthy controls and mild cases of HFMD or mild cases and severe cases of HFMD or CNS and ANS/PE or cases infected by EV71 and cases infected by CA16 based on abstract evaluation, 45 studies were selected and the full-text versions were retrieved. Of these, 8 were excluded based on the exclusion criteria. Finally, 37 studies [5], [6], [12]–[48] were included in this review. Among them, 33 studies, from Mainland China, and 4 studies, from Taiwan, addressing blood glucose and/or WBC counts in healthy controls and mild cases of HFMD, or in mild cases of HFMD and severe cases of HFMD, or in CNS and ANS/PE, or in cases infected by EV71 and cases infected by CA16 were presented in Table S1, Table S2, Table S3 and Table S4.

### Meta-regression

Using MD in the level of blood glucose between mild cases and severe cases of HFMD, we performed a meta-regression to investigate the potential interactions of age (total mean age of all cases) and gender (total number of male cases). The P-value (P<0.05) of age approached significance, which suggested that age was a confounder and may cause high between-study heterogeneity. Therefore stratified meta-analysis on blood glucose should be performed according to age.

### Meta-analysis

#### Healthy controls and mild cases of HFMD

**Figure 2:**
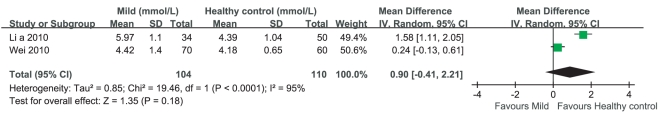
Meta-analysis of the level of blood glucose between healthy controls and mild cases of HFMD. Each comparison was presented by the name of the first author and the year of the publication. The studies were shown by a point estimate of the MD and the accompanying 95%CI which were displayed on a logarithmic scale using a random effects model. The studies are sorted according to the weight which was obtained by contribution to the pooled MD estimate. Between-study heterogeneity was tested by the x^2^-based Q-statistic, and its impact was quantified by I^2^ which can range between 0 and 100%.

There were only 2 studies pooled in this analysis. One study [13] reported that the average level of blood glucose in mild cases of HFMD was more than that in healthy control, while another study [12] claimed that there was no significant difference between them. Heterogeneity test of the 2 studies suggested that random effects model be applied (P<0.0001). According to MD calculation (Fig. 2), the average level of blood glucose in mild cases of HFMD had a 0.9 mmol/L increment compared with that in healthy controls (95%CI -0.41 mmol/L∼2.21 mmol/L). However, this effect was not statistically significant (P = 0.18).

#### Mild cases and severe cases of HFMD

**Figure 3:**
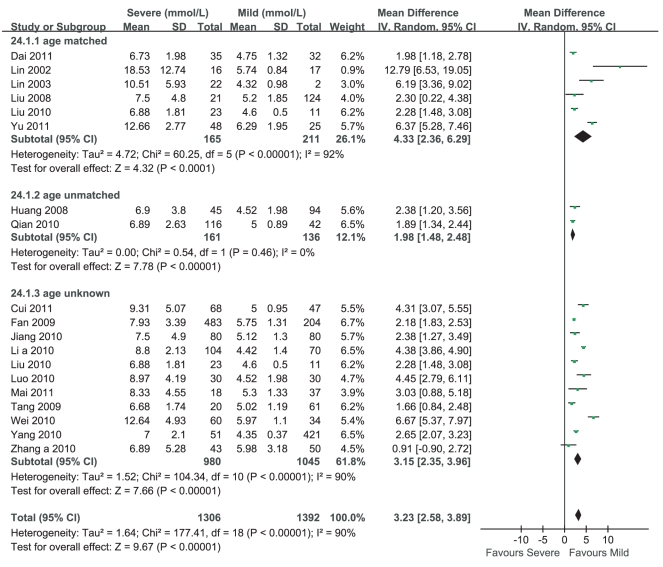
Meta-analysis of the level of blood glucose between mild cases and severe cases of HFMD. Each comparison was presented by the name of the first author and the year of the publication. The studies were shown by a point estimate of the MD and the accompanying 95%CI which were displayed on a logarithmic scale using a random effects model. The studies are sorted according to the weight which was obtained by contribution to the pooled MD estimate. Between-study heterogeneity was tested by the x^2^-based Q-statistic, and its impact was quantified by I^2^ which can range between 0 and 100%.

Eighteen studies reported that the average levels of blood glucose in severe cases of HFMD were more than those in mild cases of HFMD, while one study [23] claimed that there was no significant difference between them. Stratified analyses were performed according to the age-matched, the age-unmatched and the age-unknown. Heterogeneity test of the 19 studies suggested that random effects model be applied (P<0.000 01). According to MD calculation (Fig. 3), the meta-analyses of the age-matched, the age-unmatched and the age-unknown were consistent and the average level of blood glucose in severe cases of HFMD had a 3.10 mmol/L increment compared with that in mild cases of HFMD (95%CI 2.29 mmol/L∼3.91 mmol/L, P<0.000 01).

**Figure 4:**
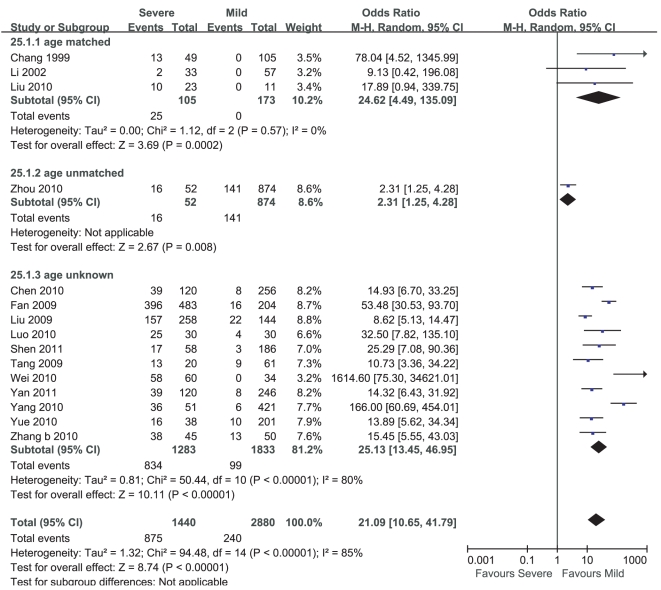
Meta-analysis of the prevalence of hyperglycemia between mild cases and severe cases of HFMD. Each comparison was presented by the name of the first author and the year of the publication. The studies were shown by a point estimate of the OR and the accompanying 95%CI which were displayed on a logarithmic scale using a random effects model. The studies are sorted according to the weight which was obtained by contribution to the pooled OR estimate. Between-study heterogeneity was tested by the x^2^-based Q-statistic, and its impact was quantified by I^2^ which can range between 0 and 100%.

Meta-analysis of the prevalence of hyperglycemia in mild cases and severe cases of HFMD included 18 studies. Stratified analyses were performed according to the age-matched, the age-unmatched and the age-unknown. Heterogeneity test of the 18 studies suggested that random effects model be applied (P<0.000 01). According to OR calculation (Fig. 4), the meta-analyses of the age-matched, the age-unmatched and the age-unknown were consistent and the prevalence of hyperglycemia in severe cases of HFMD accounted for 21.09% increment compared with that in mild cases of HFMD (95%CI 10.65%∼41.79%, P<0.000 01).

**Figure 5:**
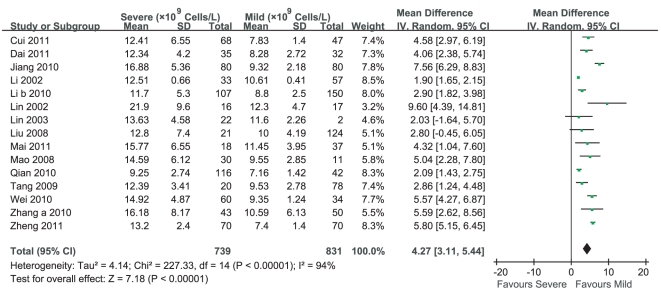
Meta-analysis of WBC counts between mild cases and severe cases of HFMD. Each comparison was presented by the name of the first author and the year of the publication. The studies were shown by a point estimate of the MD and the accompanying 95%CI which were displayed on a logarithmic scale using a random effects model. The studies are sorted according to the weight which was obtained by contribution to the pooled MD estimate. Between-study heterogeneity was tested by the x^2^-based Q-statistic, and its impact was quantified by I^2^ which can range between 0 and 100%.

Thirteen studies reported that WBC counts in severe cases of HFMD were more than those in mild cases of HFMD, while two studies [24], [30] claimed that there was no significant difference between them. Heterogeneity test of the 15 studies suggested that random effects model be applied (P<0.000 01). According to MD calculation (Fig. 5), WBC counts in severe cases of HFMD had a 4.27×10^9^Cells/L increment compared with those in mild cases of HFMD (95%CI 3.11×10^9^Cells/L∼5.44×10^9^Cells/L, P<0.000 01).

**Figure 6:**
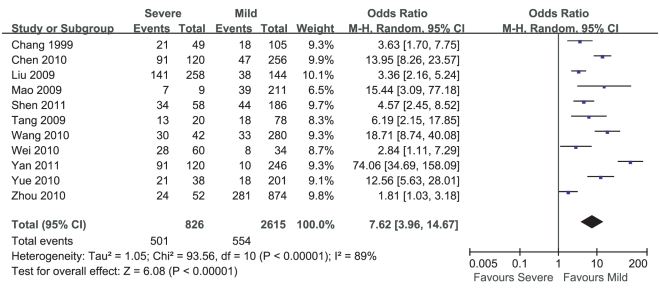
Meta-analysis of the prevalence of leukocytosis between mild cases and severe cases of HFMD. Each comparison was presented by the name of the first author and the year of the publication. The studies were shown by a point estimate of the OR and the accompanying 95%CI which were displayed on a logarithmic scale using a random effects model. The studies are sorted according to the weight which was obtained by contribution to the pooled OR estimate. Between-study heterogeneity was tested by the x^2^-based Q-statistic, and its impact was quantified by I^2^ which can range between 0 and 100%.

Meta-analysis of the prevalence of leukocytosis in mild cases and severe cases of HFMD included 11 studies. Heterogeneity test of the 11 studies suggested that random effects model be applied (P<0.000 01). According to OR calculation (Fig. 6), the prevalence of leukocytosis in severe cases of HFMD had a 7.62% increment compared with that in mild cases of HFMD (95%CI 3.96%∼14.67%, P<0.000 01).

#### CNS and ANS/PE

**Figure 7:**
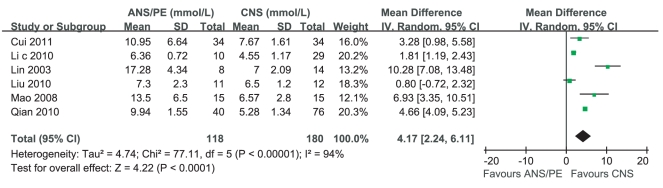
Meta-analysis of the level of blood glucose between CNS and ANS/PE. Each comparison was presented by the name of the first author and the year of the publication. The studies were shown by a point estimate of the MD and the accompanying 95%CI which were displayed on a logarithmic scale using a random effects model. The studies are sorted according to the weight which was obtained by contribution to the pooled MD estimate. Between-study heterogeneity was tested by the x^2^-based Q-statistic, and its impact was quantified by I^2^ which can range between 0 and 100%.

Five studies reported that the average levels of blood glucose in ANS/PE were more than those in CNS, while one study [18] claimed that there was no significant difference between them. Heterogeneity test of the 6 studies suggested that random effects model be applied (P<0.000 01). According to MD calculation (Fig. 7), the average level of blood glucose in ANS/PE had a 4.17 mmol/L increment compared with that in CNS (95%CI 2.24 mmol/L∼6.11 mmol/L, P<0.000 01).

**Figure 8:**
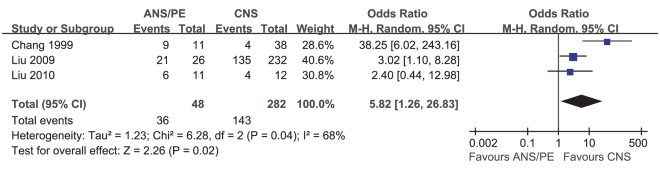
Meta-analysis of the prevalence of hyperglycemia between CNS and ANS/PE. Each comparison was presented by the name of the first author and the year of the publication. The studies were shown by a point estimate of the OR and the accompanying 95%CI which were displayed on a logarithmic scale using a random effects model. The studies are sorted according to the weight which was obtained by contribution to the pooled OR estimate. Between-study heterogeneity was tested by the x^2^-based Q-statistic, and its impact was quantified by I^2^ which can range between 0 and 100%.

Meta-analysis of the prevalence of hyperglycemia in ANS/PE and CNS of HFMD included 3 studies. Heterogeneity test of the 3 studies suggested that random effects model be applied (P = 0.04). According to OR calculation (Fig. 8), the prevalence of hyperglycemia in ANS/PE had a 5.82% increment compared with that in CNS (95%CI 1.26%∼26.83%, P = 0.02).

**Figure 9:**
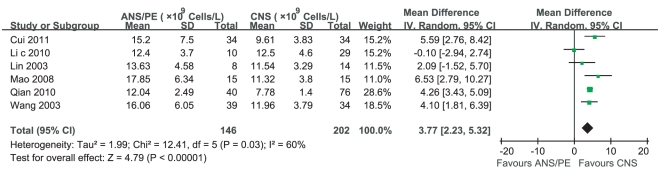
Meta-analysis of WBC counts between CNS and ANS/PE. Each comparison was presented by the name of the first author and the year of the publication. The studies were shown by a point estimate of the MD and the accompanying 95%CI which were displayed on a logarithmic scale using a random effects model. The studies are sorted according to the weight which was obtained by contribution to the pooled MD estimate. Between-study heterogeneity was tested by the x^2^-based Q-statistic, and its impact was quantified by I^2^ which can range between 0 and 100%.

Four studies reported that WBC counts in ANS/PE were more than those in CNS, while two studies [30],[43] claimed that there was no significant difference between them. Heterogeneity test of the 6 studies suggested that random effects model be applied (P = 0.03). According to MD calculation (Fig. 9), WBC counts in ANS/PE had a 3.77×10^9^Cells/L increment compared with those in CNS (95%CI 2.23×10^9^Cells/L∼ 5.32×10^9^Cells/L, P<0.000 01).

**Figure 10:**
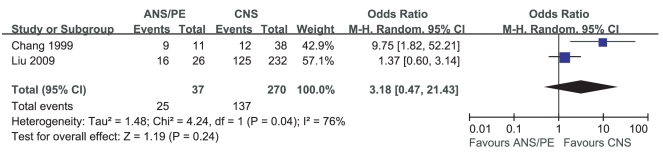
Meta-analysis of the prevalence of leukocytosis between CNS and ANS/PE. Each comparison was presented by the name of the first author and the year of the publication. The studies were shown by a point estimate of the OR and the accompanying 95%CI which were displayed on a logarithmic scale using a random effects model. The studies are sorted according to the weight which was obtained by contribution to the pooled OR estimate. Between-study heterogeneity was tested by the x^2^-based Q-statistic, and its impact was quantified by I^2^ which can range between 0 and 100%.

Meta-analysis of the prevalence of leukocytosis in CNS and ANS/PE included 2 studies. Heterogeneity test of the 2 studies suggested that random effects model be applied (P<0.04). According to OR calculation (Fig. 10), the prevalence of leukocytosis in ANS/PE had a 3.18% increment compared with that in CNS (95%CI 0.47%∼21.43%). However, this effect was not statistically significant (P = 0.24).

#### Cases infected by EV71 and cases infected by CA16

**Figure 11:**
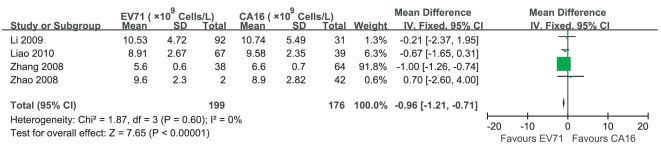
Meta-analysis of WBC counts between cases infected by EV71 and cases infected by CA16. Each comparison was presented by the name of the first author and the year of the publication. The studies were shown by a point estimate of the MD and the accompanying 95%CI which were displayed on a logarithmic scale using a random effects model. The studies are sorted according to the weight which was obtained by contribution to the pooled MD estimate. Between-study heterogeneity was tested by the x^2^-based Q-statistic, and its impact was quantified by I^2^ which can range between 0 and 100%.

One study [48] reported that WBC counts in cases infected by EV71 were less than those in cases infected by CA16, while 3 studies [45]–[47] claimed that there was no significant difference between them. Heterogeneity test of the 4 studies suggested that fixed effects model be applied (P = 0.60). According to MD calculation (Fig. 11), WBC counts in cases infected by EV71 had a 0.96×10^9^Cells/L decrement compared with those in cases infected by CA16 (95%CI 0.71×10^9^Cells/L∼1.21×10^9^Cells/L, P<0.000 01).

## Discussion

Repeated outbreaks of HFMD have occurred in many parts of the world such as Malaysia [49]–[50], Taiwan [1], [51]–[54], Singapore [55], Mainland China [56], Brunei [57], Western Australia [58], the Unites States [59] and Germany [60]. The first reported large and severe HFMD epidemic occurred in Taiwan region in 1998 including about 130 000 cases of HFMD, among whom 405 patients were severe cases and 78 died [1], [51]–[52]. In 2000, there was another report of outbreak, with 80 677 cases of HFMD and 41 deaths there [52]. In Mainland China, HFMD has occurred repeatedly in recent years and more and more cases have been reported. There were 83 344, 488 955 and 1 155 525 cases in the nationwide in the year of 2007, 2008 and 2009, respectively, reported by the Ministry of Health of the People's Republic of China. The corresponding deaths increased with years and were 17, 126 and 353 for these years, respectively. This suggested that HFMD had become a severe public health problem in China. Therefore it is necessary to find feasible indexes to monitor the severity and prognosis of HFMD patients.

When children suffer from HFMD, the prevalence of hyperglycemia, the prevalence of leukocytosis, the level of blood glucose and WBC counts will change with the severity of the disease. In this article, we performed a meta-analysis on these indexes respectively. To the best of our knowledge, this is the first time to apply meta-analysis to the prevalence of hyperglycemia, the prevalence of leukocytosis, the level of blood glucose and WBC counts in cases of HFMD.

By using a search strategy, and inclusion criteria and exclusion criteria, 37 studies were selected including 33 studies reported in Mainland China and 4 studies reported in Taiwan. Far less was known regarding the descriptive epidemiology of HFMD in countries outside the Western Pacific Region [4]. It is unfortunate that no studies reported in countries other than Mailand China and Taiwan fulfilled our inclusion criteria to be included in our analysis. Therefore our research focused on the characteristics of blood glucose and WBC counts reported in Mainland China and Taiwan. When children suffer from HFMD, the patients could present different symptoms from the mild to the severe such as HFMD, encephalitis, ANS and PE. The characteristics of blood glucose and WBC counts which have been previously reported are inconsistent. For example, one study [6] claimed that the average level of blood glucose in mild cases of HFMD was higher than that in healthy controls, but another study [5] claimed that there was no significant difference between them. As another example, some studies [8], [13], [27], [37] claimed that WBC counts in ANS/PE were higher than those in CNS but other studies [24], [38] showed that there was no significant difference between them. By meta-analysis we solved the divergence between them and concluded that the level of blood glucose, the prevalence of hyperglycemia, WBC counts and the prevalence of leukocytosis increased with the severity of the disease, but there was no significant difference in the average level of blood glucose between mild cases of HFMD and healthy controls. In the symptom-matched cases infected by EV71 and CA16, respectively, WBC counts were less in cases infected by EV71 than those in cases infected by CA16. However, there were not enough data available for meta-analysis of the level of blood glucose between cases infected by EV71 and CA16, and of WBC counts between mild cases of HFMD and healthy controls.

Blood glucose and WBC counts could help doctors manage patients in 2 ways by judging the severity and prognosis. First, blood glucose and WBC counts could reflect the severity of the illness. Lin et al [9] reported that both the level of blood glucose and WBC counts increased greatly from the mildest uncomplicated group to the severest encephalitis with pulmonary edema group when HFMD patients were divided into 3 groups: the uncomplicated group, the encephalitis group and the encephalitis with pulmonary edema group. There were also other such reports [10], [37] which suggested similar findings. Elevated level of blood glucose could also forecast disease severity. When the level of blood glucose of mild cases of HFMD increased rapidly, these mild cases would develop into severe cases in 2–4 hours [13]. Therefore doctors would have enough time to take measures in advance to manage the patients. One report denoted that when the level of blood glucose was controlled under 7.8 mmol/L the survival rate of the patients increased 5.52 times compared to that of the patients with the level of blood glucose >11.1 mmol/L [61]. Second, blood glucose and WBC counts could also be used to predict the prognosis of the illness. Elevated blood glucose and WBC counts often indicated poor prognosis. Chang et al [19] reported that all the 11 patients with blood glucose levels from 14.1 mmol/L to 42.2 mmol/L and WBC counts from 11.6×10^9^ cells/L to 40.6×10^9^ cells/L died. A patient even died 3 hours later after admission. In another report, Lin et al [24] reported that 15 patients with an average level of blood glucose of 7.66 mmol/L and WBC counts of 11.72×10^9^ cells/L all recovered after treatment while 9 patients with an average level of blood glucose of 13.84 mmol/L and WBC counts of 16.36×10^9^ cells/L all had sequelae or died. Therefore blood glucose and WBC counts were very helpful for doctors to manage HFMD patients especially in primary care settings and resource-limited settings such as most of the counties in China. These 2 indexes would also help doctors to manage HFMD patients in other countries of the world. However, they should be used combined with clinical symptoms and other detection methods such as chest x-ray, brain MRI, abnormal heart rate and Troponin I [62]–[63].

The limitations of this study should be kept in mind. First, most of the included studies were published in Chinese, quality of the reports may be variable. Second, in the included articles, the detailed information of the cases of HFMD (e.g. encephalitis, aseptic meningitis, acute flaccid paralysis, myocarditis, pulmonary edema or hemorrhage etc.) could not be obtained; relevant stratified analyses could not be performed to disclose more detailed characteristics of blood glucose and WBC counts in cases of HFMD and it was difficult to analyze the high level of heterogeneity between studies. Third, not all necessary information, such as age and gender of the study population, could be obtained from all included studies.

In conclusion, our analyses suggested that blood glucose and WBC counts increased with the severity of the illness. There was no difference in the level of blood glucose between healthy controls and mild cases of HFMD. WBC counts in cases infected by EV71 were less than those in cases infected by CA16. Our results advise monitoring of blood glucose and WBC counts in patients of HFMD, which would help doctors to manage HFMD patients greatly.

## Supporting Information

Table S1 **Characteristics of the studies on blood glucose and WBC counts in healthy controls and mild cases of HFMD considered in the meta-analysis.**
(DOC)

Table S2 **Characteristics of the studies on blood glucose and WBC counts in mild cases and severe cases of HFMD considered in the meta-analysis.**
(DOC)

Table S3 **Characteristics of the studies on blood glucose and WBC counts in CNS and ANS/PE considered in the meta-analysis.**
(DOC)

Table S4 **Characteristics of the studies on blood glucose and WBC counts in cases infected by EV71 and cases infected by CA16 considered in the meta-analysis.**
(DOC)

Table S5 **Characteristics of the studies on blood glucose and WBC counts in mild cases and severe cases of HFMD considered in the meta-regression.**
(DOC)
