# Supplementary material for: The Characteristics of Blood Glucose and WBC Counts in Peripheral Blood of Cases of Hand Foot and Mouth Disease in China: A Systematic Review
Source: PLoS One. 2012 Jan 3;7(1):e29003. doi: 10.1371/journal.pone.0029003 (PMC3250408; doi:10.1371/journal.pone.0029003)
Supplement: Table S1 — Characteristics of the studies on blood glucose and WBC counts in healthy controls and mild cases of HFMD considered in the meta-analysis. (DOC) [file pone.0029003.s001.doc]

Table S1. Characteristics of the studies on blood glucose and WBC counts in healthy controls and mild cases of HFMD considered in the meta-analysis

| study | Country/district | Selection/characteristics of healthy control | Selection/characteristics of mild cases of HFMD | The diagnosis criteria of HFMD | The diagnosis criteria of Hyperglycemia and/or Leukocytosis | Assay method | Assay time | Location rural/urban | Absence of concomitant infections | ethnicity |
| --- | --- | --- | --- | --- | --- | --- | --- | --- | --- | --- |
| Wei 2010 [12] | Sanya City, Hainan Province, China | Number: 50; Hyperglycemia: Number: 0, prevalence: 0%; Level of blood glucose: 4.39±1.04mmol/l; Leukocytosis: Number: 8, prevalence: 23.5%; WBC counts: 5.69±3.25×10^9^cells/L; N.A. | Cases: 34; Hyperglycemia: Number: 0, prevalence: 0%; Level of blood glucose: 5.97±1.10mmol/l; Leukocytosis: Number: 8, prevalence: 23.5%; WBC counts: 9.35±1.24×10^9^cells/L; N.A. | 1 | b | Blood glucose: Automatic Biochemical Analyzer;  WBC counts:  Hematology Analyzer | At admission | N.A. | N.A. | Chinese ethnicity |
| Li a 2010 [13] | Hefei City, Anhui Province, China | Cases: 60; Level of blood glucose: 4.18±0.65mmol/l; N.A. | Cases: 70; Level of blood glucose: 4.42±1.40mmol/l; N.A. | 5 | c | Blood glucose: Automatic Biochemical Analyzer; | At admission | N.A. | N.A. | Chinese ethnicity |

N.A. information was not available;

b. hyperglycemia>6.11mmol/l or random blood glucose>11.1mmol/L

c. hyperglycemia>6.11mmol/l

1 2008^th^ Handbook of prevention and control of Hand Foot and Mouth Disease issued by the Ministry of Health of the People’s Republic of China

5 2010^th^ Practical handbook of treatment of Hand Foot and Mouth Disease in children issued by Anhui Science and Technology Press.
