# Supplementary material for: The Characteristics of Blood Glucose and WBC Counts in Peripheral Blood of Cases of Hand Foot and Mouth Disease in China: A Systematic Review
Source: PLoS One. 2012 Jan 3;7(1):e29003. doi: 10.1371/journal.pone.0029003 (PMC3250408; doi:10.1371/journal.pone.0029003)
Supplement: Table S2 — Characteristics of the studies on blood glucose and WBC counts in mild cases and severe cases of HFMD considered in the meta-analysis. (DOC) [file pone.0029003.s002.doc]

Table S2. Characteristics of the studies on blood glucose and WBC counts in mild cases and severe cases of HFMD considered in the meta-analysis

| study | Country/district | Selection/characteristics of mild cases of HFMD | Selection/characteristics of severe cases of HFMD | The diagnosis criteria of HFMD | The diagnosis criteria of Hyperglycemia and/or Leukocytosis | Assay method | Assay time | Location rural/urban | Absence of concomitant infections | ethnicity |
| --- | --- | --- | --- | --- | --- | --- | --- | --- | --- | --- |
| Mao 2008 [5] | Fuyang City, Anhui Province, China | Cases: 11; Age: 36±26month; Male to female ratio: 7/4; WBC counts: 9.55±2.85×10^9^cells/L; N.A. | Cases: 30; Age: 19±11.10month; Male to female ratio: 22/8; WBC counts: 14.59±6.12×10^9^cells/L; N.A. | 1 | N.A. | N.A. | At admission | N.A. | N.A. | Chinese ethnicity |
| Yu 2011 [6] | Qingdao City, Shandong Province, China | Cases: 25; Age: 20.60±7.30month; Male to female ratio: 14/11; Level of blood glucose: 6.29±1.95mmol/l; N.A. | Cases: 48; Age: 19.18±7.59month; Male to female ratio: 27/21; Level of blood glucose: 12.66±2.77mmol/l; N.A. | N.A. | N.A. | Blood glucose: Automatic Biochemical Analyzer; | At admission | N.A. | N.A. | Chinese ethnicity |
| Wei 2010 [12] | Sanya City, Hainan Province, China | Cases: 34; Hyperglycemia: Number: 0, prevalence: 0%; Level of blood glucose: 5.97±1.10mmol/l; Leukocytosis: Number: 8, prevalence: 23.5%; WBC counts: 9.35±1.24×10^9^cells/L; N.A. | Cases: 60; Hyperglycemia: Number: 58, prevalence: 96.7%; Level of blood glucose: 12.64±4.93mmol/l; Leukocytosis: Number: 28, prevalence: 46.7%; WBC counts: 14.92±4.87×10^9^cells/L; N.A. | 1 | b | Blood glucose: Automatic Biochemical Analyzer;  WBC counts:  Hematology Analyzer | At admission | N.A. | N.A. | Chinese ethnicity |
| Li a 2010 [13] | Hefei City, Anhui Province, China | Cases: 70; Level of blood glucose: 4.42±1.40mmol/l; N.A. | Cases: 104; Level of blood glucose: 8.80±2.13mmol/l; N.A. | 5 | c | Blood glucose: Automatic Biochemical Analyzer; | At admission | N.A. | N.A. | Chinese ethnicity |
| Lou 2010 [14] | Dezhou City, Shandong Province, China | Cases: 30; Male to female ratio: 18/12; Hyperglycemia: Number: 4, prevalence: 13.3%; Level of blood glucose: 4.52±1.98mmol/l; N.A. | Cases: 30; Male to female ratio: 19/11; Hyperglycemia: Number: 25, prevalence: 83.3%; Level of blood glucose: 8.97±4.19mmol/l; N.A. | 1 | b | Blood glucose: Automatic Biochemical Analyzer; | At admission | N.A. | N.A. | Chinese ethnicity |
| Cui 2011 [15] | Zhengzhou City, Henan Province, China | Cases: 47; Level of blood glucose: 5.00±0.95mmol/l; WBC counts: 7.83±1.40×10^9^cells/L; N.A. | Cases: 68; Level of blood glucose: 9.31±5.07mmol/l; WBC counts: 12.41±6.55×10^9^cells/L; N.A | 2 | N.A. | Blood glucose: Automatic Biochemical Analyzer;  WBC counts:  Hematology Analyzer | At admission | N.A. | N.A. | Chinese ethnicity |
| Lin 2002 [16] | Taoyuan, Taiwan | Cases: 17; Age: 31±18month; Male to female ratio: 11/6; Level of blood glucose: 5.74±0.84mmol/l; WBC counts: 12.3±4.7×10^9^cells/L; N.A. | Cases: 16; Age: 23±19.09month; Male to female ratio: 8/8; Level of blood glucose: 18.53±12.74mmol/l; WBC counts: 21.9±9.60×10^9^cells/L; N.A. | According to the description of the text | N.A. | N.A. | At admission | N.A. | N.A. | Taiwanese ethnicity |
| Liu 2010 [17] | Shenzhen City, Guangdong Province, China | Cases: 11; Age: 28.40±14.60month; Hyperglycemia: Number: 0, prevalence: 0%; Level of blood glucose: 4.60±0.50mmol/l; N.A. | Cases: 23; Age: 26.29±12.31month; Hyperglycemia: Number: 10, prevalence: 43.5%; Level of blood glucose: 6.88±1.81mmol/l; N.A. | 1 | j | N.A. | At admission | N.A. | N.A. | Chinese ethnicity |
| Huang 2008 [18] | Fuyang City, Anhui Province, China | Cases: 94; Age: 39.37±20.64month; Male to female ratio: 62/32; Level of blood glucose: 4.52±1.98mmol/l; N.A. | Cases: 45; Age: 19.02±8.79month; Male to female ratio: 30/15; Level of blood glucose: 6.90±3.80mmol/l; N.A. | 1 | c | N.A. | At admission | N.A. | N.A. | Chinese ethnicity |
| Fan 2009 [19] | Hefei City, Anhui Province, China | Cases: 204; Hyperglycemia: Number: 16, prevalence: 7.84%; Level of blood glucose: 5.75±1.31mmol/l; N.A. | Cases: 483; Hyperglycemia: Number: 396, prevalence: 81.99%; Level of blood glucose: 7.93±3.39mmol/l; N.A. | 1 | c | Blood glucose: Automatic Biochemical Analyzer; | At admission | N.A. | N.A. | Chinese ethnicity |
| Yang 2010 [20] | Liuzhou City, Guangxi Province, China | Cases: 421; Male to female ratio: 215/206; Hyperglycemia: Number: 6, prevalence: 1.4%; Level of blood glucose: 4.35±0.37mmol/l; N.A. | Cases: 51; Male to female ratio: 29/23; Hyperglycemia: Number: 36, prevalence: 69.2%; Level of blood glucose: 7.00±2.10mmol/l; N.A. | 2 | c | Blood glucose : glucometer | At admission | N.A. | N.A. | Chinese ethnicity |
| Jiang 2010 [21] | Liaocheng City, Shandong Province, China | Cases: 80; Male to female ratio: 59/21; Level of blood glucose: 5.12±1.30mmol/l; WBC counts: 9.32±2.18×10^9^cells/L; N.A. | Cases: 80; Male to female ratio: 47/33; Level of blood glucose: 7.50±4.90mmol/l; WBC counts: 16.88±5.36 ×10^9^cells/L; N.A. | 3 | N.A. | N.A. | At admission | N.A. | N.A. | Chinese ethnicity |
| Li b 2010 [22] | Wenzhou City, Zhejiang Province, China | Cases: 150; Male to female ratio: 91/59; WBC counts: 8.80±2.50×10^9^cells/L; N.A. | Cases: 107; Male to female ratio: 65/42; WBC counts: 11.70±5.30×10^9^cells/L; Pathogen: 80 cases infected by EV71, 6 cases infected by CA16, 12 cases infected by non-EV71-non-CA16 and 19 detected for negative pathogens. N.A. | 4 | N.A. | N.A. | At admission | N.A. | N.A. | Chinese ethnicity |
| Zhang a 2010 [23] | Donghai County, Jiangsu Province, China | Cases: 50; Level of blood glucose: 5.98±3.18mmol/l; WBC counts: 10.59±6.13×10^9^cells/L; N.A. | Cases: 43; Level of blood glucose: 6.89±5.28mmol/l; WBC counts: 16.18±8.17×10^9^cells/L; Pathogen: 36 cases infected by EV71, 5 cases infected by CA16 and 2 cases detected for negative pathogen. N.A. | 1 | N.A. | N.A. | At admission | N.A. | N.A. | Chinese ethnicity |
| Liu 2009 [24] | Haozhou City, Anhui Province, China | Cases: 144; Hyperglycemia: Number: 22, prevalence: 15.28%; Leukocytosis: Number: 38, prevalence: 26.39%; N.A. | Cases: 258; Hyperglycemia: Number: 157, prevalence: 60.85%; Leukocytosis: Number: 141, prevalence: 54.65%; N.A. | 2 | N.A. | N.A. | At admission | N.A. | N.A. | Chinese ethnicity |
| Chang 1999 [25] | Taoyuan, Taiwan | Cases: 105; Age: 29±21month; Hyperglycemia: Number: 0, prevalence: 0%; Leukocytosis: Number: 18, prevalence: 17%; N.A. | Cases: 49; Age: 26.97±21.12month; Hyperglycemia: Number: 13, prevalence: 26.53%; Leukocytosis: Number: 21, prevalence: 42.86%; N.A. | 2 | d | N.A. | At admission | N.A. | N.A. | Taiwanese ethnicity |
| Zhang b 2010 [26] | Dongwan City, Guangdong Province, China | Cases: 50; Male to female ratio: 32/18; Hyperglycemia: Number: 13, prevalence: 26.00%; Cases with diabetes mellitus or other diseases were excluded; N.A. | Cases: 45; Male to female ratio: 28/17; Hyperglycemia: Number: 38, prevalence: 84.40%; Cases with diabetes mellitus or other diseases were excluded; N.A. | 4 | c | N.A. | At admission | N.A. | N.A. | Chinese ethnicity |
| Zhou 2010 [27] | Nantong City, Jiangsu, Province, China | Cases: 874; Age: 3.60±2.10month; Male to female ratio: 581/293; Hyperglycemia: Number: 141, prevalence: 16.13%; Leukocytosis: Number: 281, prevalence: 32.15%; N.A. | Cases: 52; Age: 2.50±2.10month; Male to female ratio: 35/17; Hyperglycemia: Number: 16, prevalence: 30.77%; Leukocytosis: Number: 24, prevalence: 78.85%; N.A. | 2 | N.A. | N.A. | At admission | N.A. | N.A. | Chinese ethnicity |
| Wang 2010 [28] | Nanyang City, Henan Province, China | Cases: 280; Leukocytosis: Number: 33, prevalence: 11.79%; N.A. | Cases: 42; Leukocytosis: Number: 30, prevalence: 71.42%; N.A. | 2 | f | WBC counts:  Hematology Analyzer | At admission | N.A. | N.A. | Chinese ethnicity |
| Chen 2010 [29] | Weifang City, Shandong Province, China | Cases: 256; Male to female ratio: 181/75; Hyperglycemia: Number: 8, prevalence: 3.20%; Leukocytosis: Number: 47, prevalence: 19.10%; N.A. | Cases: 120; Male to female ratio: 92/28; Hyperglycemia: Number: 39, prevalence: 32.50%; Leukocytosis: Number: 91, prevalence: 75.80%; N.A. | 1 | N.A. | N.A. | At admission | N.A. | N.A. | Chinese ethnicity |
| Lin 2003 [30] | Taoyuan Taiwan | Cases: 2; Age: 12.6±4.24month; Male to female ratio: 1/1; Level of blood glucose: 4.32±0.98mmol/l; WBC counts: 11.6±2.26×10^9^cells/L; N.A. | Cases: 22; Age: 22.2±15.04month; Male to female ratio: 12/10; Level of blood glucose: 10.51±5.93mmol/l; WBC counts: 13.63±4.58×10^9^cells/L; N.A. | N.A. | N.A. | N.A. | At admission | N.A. | N.A. | Taiwanese ethnicity |
| Li 2002 [31] | Taiwan, China | Cases: 57; Age: 32±20month; Male to female ratio: 28/29; Hyperglycemia: Number: 0, prevalence: 0%; WBC counts: 10.61±0.41×10^9^cells/L; N.A. | Cases: 33; Age: 25±14month; Male to female ratio: 22/11; Hyperglycemia: Number: 2, prevalence: 6.06%; WBC counts: 12.51±0.66×10^9^cells/L; N.A. | 1 | e | N.A. | At admission | N.A. | N.A. | Taiwanese ethnicity |
| Zheng 2011 [32] | Ningbo City, Zhejiang Province, China | Cases: 70; Age: 55±11month; Male to female ratio: 34/36; urban: 17, rural: 53; WBC counts: 7.4±1.4×10^9^cells/L; N.A. | Cases: 70; Age: 15±6month; Male to female ratio: 37/33; urban: 18, rural: 52; WBC counts: 13.2±2.4×10^9^cells/L; N.A. | 1 | N.A. | N.A. | At admission | N.A. | N.A. | Chinese ethnicity |
| Qian 2010 [33] | Qingdao City, Shandong Province, China | Cases: 42; Age: 47.3±13.6month; Level of blood glucose: 5.00±0.89mmol/l; WBC counts: 7.16±1.42×10^9^cells/L; N.A. | Cases: 116; Age: 30.98±13.60month; Level of blood glucose: 6.89±2.63mmol/l; WBC counts: 9.25±2.74×10^9^cells/L; N.A. | 1 | N.A. | N.A. | At admission | N.A. | N.A. | Chinese ethnicity |
| Liu 2008 [34] | Shenzhen City, Guangdong Province, China | Cases: 124; Age: 38.3±37.1month; Male to female ratio: 80/44; Level of blood glucose: 5.2±1.85mmol/l; WBC counts: 10.0±4.19×10^9^cells/L; N.A. | Cases: 21; Age: 30.1±20.6month; Male to female ratio: 11/10; Level of blood glucose: 7.5±4.8mmol/l; WBC counts: 12.8±7.4×10^9^cells/L; N.A. | 1 | N.A. | N.A. | At admission | N.A. | N.A. | Chinese ethnicity |
| Mai 2011 [35] | Nanning City, Guangxi Province, China | Cases: 37; Level of blood glucose: 5.30±1.33mmol/l; WBC counts: 11.45±3.95×10^9^cells/L; N.A. | Cases: 18; Level of blood glucose: 8.33±4.55mmol/l; WBC counts: 15.77±6.55×10^9^cells/L; N.A. | 3 | N.A. | N.A. | At admission | N.A. | N.A. | Chinese ethnicity |
| Yue 2010 [36] | Weifang City, Shandong Province, China | Number: 201; Hyperglycemia: Number: 10, prevalence: 4.98%; Leukocytosis: Number: 18, prevalence: 8.96%; N.A. | Cases: 38; Hyperglycemia: Number: 16, prevalence: 42.11%; Leukocytosis: Number: 21, prevalence: 55.26%; N.A. | 1 | g | N.A. | At admission | N.A. | N.A. | Chinese ethnicity |
| Yan 2011 [37] | Weifang City, Shandong Province, China | Number: 246; Male to female ratio: 181/75; Hyperglycemia: Number: 8, prevalence: 3.2%; Leukocytosis: Number: 10, prevalence: 4.1%; N.A. | Cases: 120; Male to female ratio: 92/48; Hyperglycemia: Number: 39, prevalence: 32.5%; Leukocytosis: Number: 91, prevalence: 75.8%; N.A. | 1 | N.A. | N.A. | At admission | N.A. | N.A. | Chinese ethnicity |
| Dai 2011 [38] | Nanzhang County, Hubei Province, China | Cases: 32; Age: 32.50±16.85month; Male to female ratio: 15/17; Level of blood glucose: 4.75±1.32mmol/l; WBC counts: 8.28±2.72×10^9^cells/L; N.A. | Cases: 35; Age: 25.46±14.80month; Male to female ratio: 17/18; Level of blood glucose: 6.73±1.98mmol/l; WBC counts: 12.34±4.20×10^9^cells/L; N.A. | 4 | N.A. | N.A. | At admission | N.A. | N.A. | Chinese ethnicity |
| Tang 2009 [39] | Liuzhou City, Guangxi Province, China | Blood glucose: total cases: 61; Hyperglycemia: Number: 9, prevalence: 14.75%; level of blood glucose: 5.02±1.19mmol/L; leukocytes: total cases: 78; Leukocytosis: Number: 18, prevalence: 23.08%; WBC counts: 9.53±2.78×10^9^cells/L; N.A. | Cases: 20; Hyperglycemia: Number: 13, prevalence: 65%; level of blood glucose: 6.68±1.74mmol/L; Leukocytosis: Number: 13, prevalence: 65%; WBC counts: 12.39±3.41×10^9^cells/L; N.A. | 7 | h | N.A. | At admission | N.A. | N.A. | Chinese ethnicity |
| Shen 2011 [40] | Ningbo City, Zhejiang Province, China | Cases: 186; Hyperglycemia: Number: 3, prevalence: 1.6%; Leukocytosis: Number: 44, prevalence: 23.7%; N.A. | Cases: 58; Hyperglycemia: Number: 17, prevalence: 29.3%; Leukocytosis: Number: 34, prevalence: 58.6%; N.A. | 5 | i | N.A. | At admission | N.A. | N.A. | Chinese ethnicity |
| Mao 2009 [41] | Hangzhou City, Zhejiang Province, China | Cases: 211; Leukocytosis: Number: 39, prevalence: 18.48%; N.A. | Cases: 9; Leukocytosis: Number: 7, prevalence: 77.78%; N.A. | 1 | N.A. | N.A. | At admission | N.A. | N.A. | Chinese ethnicity |

N.A. information was not available;

b. hyperglycemia>6.11mmol/l or random blood glucose>11.1mmol/L

c. hyperglycemia>6.11mmol/l

d. hyperglycemia>8.3mmol/l, leukocytosis >17.5×10^9^cells/L

e. hyperglycemia>8.3mmol/l

f. leukocytosis >10×10^9^cells/L

g. hyperglycemia: N.A. leukocytosis >14×10^9^cells/L

h. hyperglycemia>6.11mmol/l, leukocytosis: N.A.

j hyperglycemia>7.0mmol/l

1 2008^th^ Handbook of prevention and control of Hand Foot and Mouth Disease issued by the Ministry of Health of the People’s Republic of China

2 Handbook of treatment of enterovirus (EV71) issued by the Ministry of Health of the People’s Republic of China

3 2009^th^ Handbook of prevention and control of Hand Foot and Mouth Disease issued by the Ministry of Health of the People’s Republic of China

4 2010^th^ Handbook of prevention and control of Hand Foot and Mouth Disease issued by the Ministry of Health of the People’s Republic of China

5 2010^th^ Practical handbook of treatment of Hand Foot and Mouth Disease in children issued by Anhui Science and Technology Press.

7 The latest scientific theories and practice of skin written by Zhang Kaiming, Wang Gang, Yi Guohua. China Medical Science and Technology Publishing House. 2001: 121-122.
