# Supplementary material for: The Characteristics of Blood Glucose and WBC Counts in Peripheral Blood of Cases of Hand Foot and Mouth Disease in China: A Systematic Review
Source: PLoS One. 2012 Jan 3;7(1):e29003. doi: 10.1371/journal.pone.0029003 (PMC3250408; doi:10.1371/journal.pone.0029003)
Supplement: Table S3 — Characteristics of the studies on blood glucose and WBC counts in CNS and ANS/PE considered in the meta-analysis. (DOC) [file pone.0029003.s003.doc]

Table S3. Characteristics of the studies on blood glucose and WBC counts in CNS cases and ANS/PE cases of HFMD considered in the meta-analysis

| study | Country/district | Selection/characteristics of CNS cases of HFMD | Selection/characteristics of ANS/PE cases of HFMD | The diagnosis criteria of HFMD | The diagnosis criteria of Hyperglycemia and/or Leukocytosis | Assay method | Assay time | Location rural/urban | Absence of concomitant infections | ethnicity |
| --- | --- | --- | --- | --- | --- | --- | --- | --- | --- | --- |
| Mao 2008 [5] | Fuyang City, Anhui Province, China | Cases: 15; Age: 23±11month; Male to female ratio: 13/2; Level of blood glucose: 6.57±2.80mmol/l; WBC counts: 11.32±3.80×10^9^cells/L; N.A. | Cases: 15; Age: 15±10month; Male to female ratio: 9/6; Level of blood glucose: 13.50±6.50mmol/l; WBC counts: 17.85±6.34×10^9^cells/L; N.A. | 1 | N.A. | N.A. | At admission | N.A. | N.A. | Chinese ethnicity |
| Cui 2011 [15] | Zhengzhou City, Henan Province, China | Cases: 34; Level of blood glucose: 7.67±1.61mmol/l; WBC counts: 9.61±3.83×10^9^cells/L; N.A. | Cases: 34; Level of blood glucose: 10.95±6.64mmol/l; WBC counts: 15.20±7.50×10^9^cells/L; N.A | 2 | N.A. | Blood glucose: Automatic Biochemical Analyzer;  WBC counts:  Hematology Analyzer | At admission | N.A. | N.A. | Chinese ethnicity |
| Liu 2010 [17] | Shenzhen City, Guangdong Province, China | Cases: 12; Age: 30.5±13.8month; Hyperglycemia: Number: 4, prevalence: 33.3%; Level of blood glucose: 6.5±1.2mmol/l; N.A. | Cases: 11; Age: 21.7±8.9month; Hyperglycemia: Number: 6, prevalence: 54.5%; Level of blood glucose: 7.3±2.3mmol/l; N.A. | N.A. | j | N.A. | At admission | N.A. | N.A. | Chinese ethnicity |
| Liu 2009 [24] | Haozhou City, Anhui Province, China | Cases: 232; Hyperglycemia: Number: 135, prevalence: 58.19%; Leukocytosis: Number: 125, prevalence: 53.88%; N.A. | Cases: 26; Hyperglycemia: Number: 21, prevalence: 80.77%; Leukocytosis: Number: 16, prevalence: 61.54%; N.A. | 2 | N.A. | N.A. | At admission | N.A. | N.A. | Chinese ethnicity |
| Chang 1999 [25] | Taoyuan, Taiwan | Cases: 38; Age: 29±21month; Hyperglycemia: Number: 4, prevalence: 11%; Leukocytosis: Number: 12, prevalence: 32%; N.A. | Cases: 11; Age: 20±21.month; Hyperglycemia: Number: 9, prevalence: 82%; Leukocytosis: Number: 9, prevalence: 82%; N.A. | 2 | d | N.A. | At admission | N.A. | N.A. | Taiwanese ethnicity |
| Lin 2003 [30] | Taoyuan Taiwan | Cases: 14; Age: 24.86±17.46month; Male to female ratio: 8/6; Level of blood glucose: 7±2.09mmol/l; WBC counts: 11.54±3.29×10^9^cells/L; N.A. | Cases: 8; Age: 17.55±8.56month; Male to female ratio: 4/4; Level of blood glucose: 17.28±4.34mmol/l; WBC counts: 13.63±4.58×10^9^cells/L; N.A. | N.A. | N.A. | N.A. | At admission | N.A. | N.A. | Taiwanese ethnicity |
| Qian 2010 [33] | Qingdao City, Shandong Province, China | Cases: 76; Age: 31.5±12.3month; Level of blood glucose: 5.28±1.34mmol/l; WBC counts: 7.78±1.40×10^9^cells/L; N.A. | Cases: 40; Age: 30.0±15.9month; Level of blood glucose: 9.94±1.55mmol/l; WBC counts: 12.04±2.49×10^9^cells/L; N.A. | 1 | N.A. | N.A. | At admission | N.A. | N.A. | Chinese ethnicity |
| Wang 2003 [42] | Tainan and Taipei, Taiwan | Cases: 34; Age: 33.6±39.1month; WBC counts: 11.96±3.79×10^9^cells/L; N.A. | Cases: 39; Age: 19.35±14.19month; WBC counts: 16.06±6.05×10^9^cells/L; N.A. | N.A. | N.A. | N.A. | At admission | N.A. | N.A. | Taiwanese ethnicity |
| Li c 2010 [43] | Wenzhou City, Zhejiang Province, China | Cases: 29; Level of blood glucose: 4.55±1.17mmol/l; WBC counts: 12.5±4.6×10^9^cells/L;N.A. | Cases: 10; Level of blood glucose: 6.36±0.72mmol/l; WBC counts: 12.4±3.7×10^9^cells/L; N.A. | N.A. | N.A. | N.A. | At admission | N.A. | N.A. | Chinese ethnicity |

N.A. information was not available;

CNS: HFMD cases with central nervous system complications; ANS: HFMD cases with autonomic nervous system dysregulation; PE: HFMD cases with pulmonary edema.

d. hyperglycemia>8.3mmol/l, leukocytosis >17.5×10^9^cells/L

j hyperglycemia>7.0mmol/l

1 2008^th^ Handbook of prevention and control of Hand Foot and Mouth Disease issued by the Ministry of Health of the People’s Republic of China

2 Handbook of treatment of enterovirus (EV71) issued by the Ministry of Health of the People’s Republic of China
