# Supplementary material for: The Characteristics of Blood Glucose and WBC Counts in Peripheral Blood of Cases of Hand Foot and Mouth Disease in China: A Systematic Review
Source: PLoS One. 2012 Jan 3;7(1):e29003. doi: 10.1371/journal.pone.0029003 (PMC3250408; doi:10.1371/journal.pone.0029003)
Supplement: Table S4 — Characteristics of the studies on blood glucose and WBC counts in cases infected by EV71 and cases infected by CA16 considered in the meta-analysis. (DOC) [file pone.0029003.s004.doc]

Table S4. Characteristics of the studies on blood glucose and WBC counts in HFMD patients infected by EV71 and CA16 considered in the meta-analysis

| study | Country/district | Selection/characteristics of HFMD patients infected by EV71 | Selection/characteristics of HFMD patients infected by CA16 | The diagnosis criteria of HFMD | The diagnosis criteria of Hyperglycemia and/or Leukocytosis | Assay method | Assay time | Location rural/urban | Absence of concomitant infections | ethnicity |
| --- | --- | --- | --- | --- | --- | --- | --- | --- | --- | --- |
| Li d 2010 [44] | Linyi City, Shandong Province, China | Number: 63; Hyperglycemia: Number: 10, prevalence: 15.87%; Leukocytosis: Number: 30, prevalence: 47.62%; Body temperature and duration have no statistical significance  with those of CA16; 39 cases with vesicles in hand foot and mouth, 14 in hand and foot, and 10 without vesicles; 41 cases with central nervous system: 1 with headache, 23 with vomiting, 20 with drowsiness, 26 with tremble, 26 with convulsion, 1 with twitch, 2 with eye adjustment disorder, 4 with limb weakness, 5 with irregular breathing; N.A. | Cases: 16; Hyperglycemia: Number: 1, prevalence: 6.25%; Leukocytosis: Number: 5, prevalence: 31.25%; 10 cases with vesicles in hand foot and mouth, 5 in hand and foot, and 1 without vesicles; 16 cases with central nervous system: 4 with headache, 6 with vomiting, 3 with drowsiness, 3 with convulsion, 2 with twitch, 1 with irregular breathing; N.A. | 1 | d | N.A. | At admission | N.A. | N.A. | Chinese ethnicity |
| Liao 2010 [45] | Shaoguan City, Guangdong Province, China | Cases: 67; WBC counts: 8.91±2.67×10^9^cells/L; N.A. | Cases: 39; WBC counts: 9.58±2.35×10^9^cells/L; N.A. | 1 | N.A. | N.A. | At admission | N.A. | N.A. | Chinese ethnicity |
| Li 2009 [46] | Shenzhen City, Guangdong Province, China | Cases: 92; Age: 30.36±24.59month; Level of blood glucose: 5.85±2.62mmol/l; WBC counts: 10.53±4.72×10^9^cells/L; Body Temperature: 38.76±0.94℃, Duration: 3.70±1.90d; NP(%): 54.70±14.76; CRP: positive: 18 cases; CNSC: 31; MJOL: 31; N.A. | Cases: 31; Age: 31.61±19.99month; Level of blood glucose: 5.41±1.18mmol/l; WBC counts: 10.74±5.49×10^9^cells/L; Body Temperature: 38.36±1.07℃, Duration: 2.39±1.45d; NP(%): 48.64±14.97; CRP: positive: 7 cases; CNSC: 3; MJOL: 4; N.A. | 1＆6 | N.A. | N.A. | At admission | N.A. | N.A. | Chinese ethnicity |
| Zhao 2008 [47] | Beijing City, China | Cases: 2; WBC counts: 9.60±2.30×10^9^cells/L; fever: 2 cases; 1 cases with vesicles in the buttock, elbow and perianas, Eruption: 1.00±0.00d, Duration: 5.50±0.50d; N.A. | Cases: 42; WBC counts: 8.90±2.82×10^9^cells/L; fever: 39 cases; 21 cases with vesicles in the buttock, elbow and perianas, Eruption: 1.14±0.26d, Duration: 5.40±1.12d; N.A. | 6 | N.A. | N.A. | At admission | N.A. | N.A. | Chinese ethnicity |
| Zhang 2008 [48] | Shenzhen City, Guangdong Province, China | Cases: 38; Age: 50.4±4.8month; WBC counts: 5.6±0.6×10^9^cells/L; Body Temperature:37.6±0.5℃, Duration of fever: 2.2±0.6d; 38 cases with vesicles in hand, foot and mouth, Duration: 7.1±1.6d; 3 cases with complications; No deaths; N.A. | Cases: 64; Age: 50.4±3.6month; WBC counts: 6.6±0.7×10^9^cells/L; Body Temperature:37.7±0.3℃, Duration of fever: 2.1±0.7d; 64 cases with vesicles in hand, foot and mouth, Duration: 6.6±0.9d; 3 cases with complications; No deaths; N.A. | N.A. | N.A. | N.A. | At admission | N.A. | N.A. | Chinese ethnicity |

N.A. information was not available;

NP: neutrophils percentage; CRP: C-reactive protein; CNSC: central nervous system complication; MJOL: myoclonic jerk of limb

d. hyperglycemia>8.3mmol/l, leukocytosis >17.5×10^9^cells/L

1 2008^th^ Handbook of prevention and control of Hand Foot and Mouth Disease issued by the Ministry of Health of the People’s Republic of China

6 7^th^ Practical handbook of treatment of Hand Foot and Mouth Disease in children issued by People’s Medical Publishing House.
