# Supplementary material for: The Characteristics of Blood Glucose and WBC Counts in Peripheral Blood of Cases of Hand Foot and Mouth Disease in China: A Systematic Review
Source: PLoS One. 2012 Jan 3;7(1):e29003. doi: 10.1371/journal.pone.0029003 (PMC3250408; doi:10.1371/journal.pone.0029003)
Supplement: Table S5 — Characteristics of the studies on blood glucose and WBC counts in mild cases and severe cases of HFMD considered in the meta-regression. (DOC) [file pone.0029003.s005.doc]

Table S5 Characteristics of the studies on blood glucose and WBC counts in mild cases and severe cases of HFMD considered in the meta-regression

| study | Country/district | Selection/characteristics of mild cases of HFMD | Selection/characteristics of severe cases of HFMD | The diagnosis criteria of HFMD | The diagnosis criteria of Hyperglycemia and/or Leukocytosis | Assay method | Assay time | Location rural/urban | Absence of concomitant infections | ethnicity |
| --- | --- | --- | --- | --- | --- | --- | --- | --- | --- | --- |
| Yu 2011 [6] | Qingdao City, Shandong Province, China | Cases: 25; Age: 20.60±7.30month; Male to female ratio: 14/11; Level of blood glucose: 6.29±1.95mmol/l; N.A. | Cases: 48; Age: 19.18±7.59month; Male to female ratio: 27/21; Level of blood glucose: 12.66±2.77mmol/l; N.A. | N.A. | N.A. | Blood glucose: Automatic Biochemical Analyzer; | At admission | N.A. | N.A. | Chinese ethnicity |
| Lin 2002 [16] | Taoyuan, Taiwan | Cases: 17; Age: 31±18month; Male to female ratio: 11/6; Level of blood glucose: 5.74±0.84mmol/l; WBC counts: 12.3±4.7×10^9^cells/L; N.A. | Cases: 16; Age: 23±19.09month; Male to female ratio: 8/8; Level of blood glucose: 18.53±12.74mmol/l; WBC counts: 21.9±9.60×10^9^cells/L; N.A. | According to the description of the text | N.A. | N.A. | At admission | N.A. | N.A. | Taiwanese ethnicity |
| Huang 2008 [18] | Fuyang City, Anhui  Province, China | Cases: 94; Age: 39.37±20.64month; Male to female ratio: 62/32; Level of blood glucose: 4.52±1.98mmol/l; N.A. | Cases: 45; Age: 19.02±8.79month; Male to female ratio: 30/15; Level of blood glucose: 6.90±3.80mmol/l; N.A. | 1 | c | N.A. | At admission | N.A. | N.A. | Chinese ethnicity |
| Lin 2003 [30] | Taoyuan Taiwan | Cases: 2; Age: 12.6±4.24month; Male to female ratio: 1/1; Level of blood glucose: 4.32±0.98mmol/l; WBC counts: 11.6±2.26×10^9^cells/L; N.A. | Cases: 22; Age: 22.2±15.04month; Male to female ratio: 12/10; Level of blood glucose: 10.51±5.93mmol/l; WBC counts: 13.63±4.58×10^9^cells/L; N.A. | N.A. | N.A. | N.A. | At admission | N.A. | N.A. | Taiwanese ethnicity |
| Liu 2008 [34] | Shenzhen City, Guangdong Province, China | Cases: 124; Age: 38.3±37.1month; Male to female ratio: 80/44; Level of blood glucose: 5.2±1.85mmol/l; WBC counts: 10.0±4.19×10^9^cells/L; N.A. | Cases: 21; Age: 30.1±20.6month; Male to female ratio: 11/10; Level of blood glucose: 7.5±4.8mmol/l; WBC counts: 12.8±7.4×10^9^cells/L; N.A. | 1 | N.A. | N.A. | At admission | N.A. | N.A. | Chinese ethnicity |
| Dai 2011 [38] | Nanzhang County, Hubei Province, China | Cases: 32; Age: 32.50±16.85month; Male to female ratio: 15/17; Level of blood glucose: 4.75±1.32mmol/l; WBC counts: 8.28±2.72×10^9^cells/L; N.A. | Cases: 35; Age: 25.46±14.80month; Male to female ratio: 17/18; Level of blood glucose: 6.73±1.98mmol/l; WBC counts: 12.34±4.20×10^9^cells/L; N.A. | 4 | N.A. | N.A. | At admission | N.A. | N.A. | Chinese ethnicity |

N.A. information was not available;

c. hyperglycemia>6.11mmol/l

1 2008^th^ Handbook of prevention and control of Hand Foot and Mouth Disease issued by the Ministry of Health of the People’s Republic of China

4 2010^th^ Handbook of prevention and control of Hand Foot and Mouth Disease issued by the Ministry of Health of the People’s Republic of China
